# Birth and developmental correlates of birth weight in a sample of children with potential sensory processing disorder

**DOI:** 10.1186/1471-2431-13-29

**Published:** 2013-02-25

**Authors:** Simone V Gill, Teresa A May-Benson, Alison Teasdale, Elizabeth G Munsell

**Affiliations:** 1 Department of Occupational Therapy, Boston University, College of Health and Rehabilitation Sciences: Sargent College, 635 Commonwealth Avenue, 02215, Boston, MA, USA; 2 The Spiral Foundation, Newton, MA, USA; 3 OTA The Koomar Center, Newton, MA, USA

**Keywords:** Development, Birth weight, Child

## Abstract

**Background:**

Most research examining birth history (i.e. related birth complications) and developmental milestone achievement follow outcomes for infants at-risk with very specific birth weight categories and gestational age classifications. The purpose of this study was to examine how birth weight relates to infants’ birth histories and developmental milestone achievement when they fall into a variety of birth weight and gestational age categories.

**Methods:**

In the current study, we examined birth histories and onset ages for developmental milestones by analyzing a convenience sample of anonymous existing data from 663 developmental histories completed by parents at the time of an initial evaluation at a pediatric outpatient occupational therapy clinic. Infants fell into 3 birth weight categories; low birth weight (LBW), normal birth weight (NBW), and high birth weight (HBW) and 3 gestational age classifications considered with birth weight; small for gestational age (SGA), appropriate for gestational age (AGA), and large for gestational age (LGA).

**Results:**

NBW, AGA, and SGA infants with related birth complications had lower birth weights than infants without birth complications. Larger birth weights were associated with earlier ages for independent sitting for HBW infants, earlier ages for eating solids for NBW infants, and earlier walking onsets for LBW and NBW infants. Higher birth weights were also linked with rolling at a younger age for LGA infants, earlier walking and speaking words for AGA infants, and sooner independent sitting for SGA and AGA infants.

**Conclusions:**

Our findings suggest that birth weight and gestational age categories provide unique insights into infants’ birth history and developmental milestone achievement.

## Background

Infants’ weight at birth is a strong predictor of neonatal health outcomes such as chances of survival, risk of medical complications, and timing for the achievement of development milestones [1]. The health risks associated with birth weight depend upon classifications defined by the Centers for Disease Control [2]; extremely low birth weight (ELBW), very low birth weight (VLBW), low birth weight (LBW), normal birth weight (NBW), and high birth weight (HBW). Infants < 1,000 g, < 1,500 g, < 2,500 g, < 4,000 g, and > 4,000 g are classified as ELBW, VLBW, LBW, NBW, or HBW respectively. Weights that are ELBW, VLBW, or LBW are associated with complications such as hypothermia, hypoglycemia, perinatal asphyxia, respiratory distress, anemia, impaired nutrition, infection, neurological trouble, and hearing deficits [3-6]. Neonatal complications increase markedly depending on infants’ birth weight classifications; the less infants weigh, the higher the chances of encountering health difficulties.

When determining neonatal health outcomes, it is important to consider infants’ gestational age along with birth weight. For example, ELBW infants’ survival chances are related to gestational age: fewer weeks of gestation correlate with lower chances of survival [7]. Also, it is possible for infants with LBW to be either appropriate or small for their gestational age. Infants can be classified according to how typical their weights are for their gestational age compared to infants of the same sex [2]. Infants below the 10th percentile are small for gestational age (SGA), those between the 10th and 90th percentile are appropriate for gestational age (AGA), and infants above the 90th percentile are considered to be large for gestational age (LGA). Some health risks for SGA infants include perinatal asphyxia, sepsis, hypothermia, apnea, and risks of infection [8] whereas risks for LGA infants are shoulder dystocia [9], brachial plexus injuries [10], or hypoxic injuries [11]. Premature births are usually associated with related health outcomes [12-14] in addition to birth weight category or gestational age classification. Even late pre-term infants (LPT) born between 34 and 36 weeks gestation have recently been earmarked as having difficulty with executive function in their preschool years [15].

Most research examining health and developmental outcomes based on birth weight and gestational age follow outcomes for infants at-risk with very specific birth weight categories and gestational age classifications (i.e. ELBW, VLBW, LBW or SGA infants) into infancy [3-5,8-11]. Few have looked at outcomes with a group of infants that fall into varied birth weight categories and gestational age classifications. The purpose of this study was to examine possible associations between birth weight and birth history (i.e. related birth complications) as well as developmental milestone achievement in a sample of children with or suspected of having sensory processing disorder.

## Method

Data for this study were collected as part of a larger study with a total of 1,000 responses, which examined the incidence of pre-, peri- and post-natal factors in children identified with sensory processing problems. In the current retrospective study, participants were a convenience sample of 663 children between 4–12 years of age who received a comprehensive sensory integration-based occupational therapy evaluation at a private pediatric outpatient occupational therapy clinic (OTA-Watertown). This study is unique in that it examines the issue of birth weight and related milestone development in a population of children with sensory processing problems.

In the current study, we only included participants with recorded birth weights. Participants were primarily Caucasian and from a middle to upper-middle socio-economic status. The sample included 74% boys and 26% girls. This study had a disproportionate number of boys because boys are significantly more likely to have sensory processing problems and thus seek clinical services.

Data for this study were responses to questions on the OTA-Watertown Developmental/Sensory History completed by parents at the time of the participants’ evaluation. The developmental/sensory histories were mailed to parents generally two weeks prior to the evaluation. Parents completed the developmental/sensory histories at home and returned them to OTA-Watertown via mail prior to the evaluation session or in person on the day of the evaluation. In the current study, we analyzed parents’ anonymous, de-identified responses to questions related to their children’s birth history and developmental milestones. Milestones for children born at LBW and ELBW were corrected for prematurity. Gestational age classifications were based on growth charts. Inclusion criteria were that the developmental/sensory histories be completed between July 2001 and October 2010 and that children be between 4 and 12 years old. All developmental histories during this time period were considered for inclusion in this study. Exclusion criteria were that children not be diagnosed with Autism or Downs Syndrome. See Table 1 for specific questions used in the current study. Parents’ responses to questions on the developmental/sensory history forms were coded (i.e. Parents’ responses were converted to numerical values to be entered into statistical software) and analyzed to quantify parents’ responses. Oversight for this study was provided by the Spiral Foundation and Boston University Institutional Review Boards.

**Table 1:** Questions from developmental/sensory histories

**I. Child’s birth history: Parents were asked to check a box labeled yes, no, or don’t know to answer “Was or did the child”:**	**II. Developmental milestones: Parents were asked to provide ages for the following:**
1) Full term? Here they also wrote down the child’s birth weight.	1) rolling over
2) Premature? Here they also wrote down the number of weeks.	2) walking
3) Small for gestational age (SGA)?	3) saying words
4) Breech (feet first)?	4) sitting alone
5) Require forceps for delivery?	5) chewing solid food
6) Require suction for delivery?	6) saying sentences
7) Have any birth injuries? Here parents were asked to provide a description.	7) crawling
8) If known, Apgar score at 1 minute and at 5 minutes.	
9) Require intensive care hospitalization? Here they also answered if yes, for how long? Note that intensive care hospitalization was the neonatal intensive care unit (NICU).	

NOTE: The variable that coded whether children required suction for delivery was excluded from the analyses to avoid ambiguity about whether parents were responding with regard to suction as a clearing of the mouth and airway or as vacuum assisted delivery.

### Analyses

All statistical analyses were conducted using SPSS 16.0 statistical software. Birth weight was the main dependent variable. The independent variables were: 1) birth weight category, 2) gestational age classification considered with birth weight, and 3) birth history. The results were presented as means and standard deviations. Independent t-tests were conducted to analyze the influence of the independent variables on the dependent variable. Separate Pearson’s correlations for birth weight categories and gestational age classifications considered with birth weight were conducted to examine relationships between the age at which developmental milestones were achieved and birth weight. To test whether parents’ responses differed according to their children’s age when developmental histories were completed, a Pearson’s correlation was run to examine the relationship between children’s age at evaluation and each variable. All tests were subjected to Bonferroni corrections to reduce experiment-wise errors.

## Results

### Descriptive information

Our sample of 663 included ELBW (*n* = 3), VLBW (*n* = 8), LBW (*n* = 39), NBW (*n* = 513), and HBW (*n* = 100) children. Of the 663 in our sample, 91 were SGA, 509 were AGA, and 63 were LGA. Tables 2 and 3 show infants’ average birth weights based on birth weight category and gestational age classification considered with birth weight.

**Table 2:** Birth weight means and standard deviations by birth weight category

**Birth weight categories**	**Means in grams (standard deviations)**
ELBW	831.57 (182.26)
VLBW	1268.55 (169.37)
LBW	2208.68 (252.60)
NBW	3391.02 (380.50)
HBW	4302.95 (283.58)

**Table 3:** Birth weight means and standard deviations by gestational age classification considered with birth weight

**Gestational age classification considered with birth weight**	**Means in grams (standard deviations)**
SGA	2300.94 (532.90)
AGA	3500.68 (365.39)
LGA	4403.77 (308.53)

Although the total sample included 663, a variable number of responses were gathered due to missing data. Missing data occurred because parents sometimes left items blank on the mailed developmental histories. If items were left blank for a particular dependent variable, those infants were omitted from the analyses for that variable. For birth history, ELBW and VLBW groups were excluded from the analyses due to small *n’*s (*n* = 3 and *n* = 8 respectively). However, all LBW, NBW, and HBW infants contributed data for birth history. For developmental milestones, responses varied based on how many parents provided a specific age at which the milestone was achieved. For example, instead of listing the exact age at which the milestone was achieved or leaving the item blank, some parents wrote whether their infant achieved the milestone at an early, normal, or late age. For rolling, walking, saying words, independent sitting, eating solids, speaking sentences, and crawling this accounted for 11%, 4%, 11%, 10%, 11%, 12%, and 10% of the responses respectively. These responses were excluded from the analyses to allow for exact ages to be used rather than qualitative estimations of milestone achievement. Similar to related birth complications, ELBW and VLBW infants were excluded because of the small n for those groups. Related birth complications that were reported by birth weight category were: for LBW children a swollen head, for NBW children head bruises, face or head abrasions, broken clavicles, shoulder dystocia, a swollen head, umbilical cord around the neck, minocin, low muscle tone, dislocated hip, effusion of the forehead or nose, cranial bleeding, eye irritation, a benign mass on the neck, or a hernia, and for HBW children head bruises, face or head abrasions, broken clavicles, shoulder dystocia, low muscle tone, or cyanosis. According to gestational age classification considered with birth weight, reported related birth complications included for SGA head bruising, a swollen head, and the umbilical cord around the neck, for AGA head bruises, face or head abrasions, broken clavicles, shoulder dystocia, a swollen head, umbilical cord around the neck, minocin, low muscle tone, dislocated hip, effusion of the forehead or nose, cranial bleeding, eye irritation, a benign mass on the neck, or a hernia, and for LGA head bruises, face or head abrasions, broken clavicles, shoulder dystocia, low muscle tone, or cyanosis.

### Birth history

We examined whether birth weight is associated with related birth complications. Tables 4 and 5 show birth weight means for children with and without related birth complications based on birth weight category and gestational age classification considered with birth weight. With birth weight category as the independent variable, results were only significant for NBW infants; NBW infants with birth complications had lower birth weights than infants without complications; *t*(511) = 3.92, *p* = .0001. Of the infants with normal birth weights and birth complications 16% were premature, 5% were breech, 25% required forceps, 6% sustained birth injuries, 24% had jaundice, and 8% spent time in intensive care. Analyses with gestational age classification considered with birth weight as the independent variable showed that infants who were SGA had lower birth weights if they had related birth complications compared to SGA infants with no birth complications; *t*(89) = 2.47, *p* = .016. Of the SGA infants with birth complications 73% were premature, 8% were breech, 25% required forceps, 8% sustained birth injuries, 46% had jaundice, and 42% spent time in intensive care. We also found that AGA infants with birth complications had lower birth weights than AGA infants with none; *t*(507) = 2.35, *p* = .019. For AGA infants who had birth complications, 11% were premature, 5% were breech, 24% required forceps, 5% sustained birth injuries, 23% had jaundice, and 7% spent time in intensive care. Pearson’s correlations between birth weight and children’s age at evaluation were not significant for children with and without related birth complications (all *p’*s > .05).

**Table 4:** Birth weight means and standard deviations by birth weight category and related birth complications

**Birth weight categories**	**Birth complications factors (Y or N)**	**Prevalence of birth complications**	**Means in grams (standard deviations)**
ELBW	Yes	100%	831.57 (182.26)
	No	N/A	N/A
VLBW	Yes	100%	1268.55 (169.37)
	No	N/A	N/A
LBW	Yes	95%	2205.48 (259.96)
	No	N/A	2267.92 (200.46)
NBW	Yes	52%	3329.19 (408.73)
	No	N/A	3459.19 (334.53)
HBW	Yes	44%	4305.50 (278.86)
	No	N/A	4300.95 (289.73)

**Table 5:** Birth weight means and standard deviations by gestational age classification considered with birth weight and related birth complications

**Gestational age classification considered with birth weight**	**Birth complications factors (Y or N)**	**Prevalence of birth complications**	**Means in grams (standard deviations)**
SGA	Yes	88%	2251.26 (542.87)
	No	N/A	2667.23 (253.71)
AGA	Yes	50%	3462.68 (379.44)
	No	N/A	3538.54 (347.45)
LGA	Yes	43%	4430.32 (289.54)
	No	N/A	4383.86 (324.64)

### Developmental milestones

We examined the relationship between the age at which children achieved developmental milestones (i.e. rolling, sitting independently, crawling, walking, eating solids, speaking words, and speaking sentences) and their birth weight as infants. Tables 6 and 7 shows means for age at onset for milestones based on birth weight category and gestational age classification considered with birth weight and the number of infants that contributed to the findings for each.

**Table 6:** Birth weight means and standard deviations for each developmental milestone onset age by birth weight category

**Birth weight category**	**Developmental milestone**	**Mean onset age in months (standard deviations); n**	**# Premature; # late pre-term (34 to 36 weeks)**	**Birth weight means in grams (standard deviations)**
ELBW	Rolling	5.50 (3.53); 0	2; 0	765.42 (200.46)
	Sitting	8.00 (1.41); 0	2; 0	
	Crawling	10.50 (0.71); 0	3; 0	
	Walking	13.00 (1.73); 0	3; 0	
	Solids	10.50 (2.12); 0	2; 0	
	Words	24.00 (8.49); 0	2; 0	
	Sentences	54.00 (0); 0	1; 0	
VLBW	Rolling	3.80 (0.84); 0	5; 1	1264.37 (157.33)
	Sitting	8.00 (2.16); 0	4; 0	
	Crawling	9.58 (2.29); 0	6; 1	
	Walking	14.75 (2.25); 0	7; 1	
	Solids	9.67 (1.15); 0	3; 0	
	Words	15.79 (8.64); 0	6; 1	
	Sentences	24.60 (12.80); 0	4; 0	
LBW	Rolling	5.55 (2.34);19	16; 8	2309.70 (193.98)
	Sitting	7.62 (1.62); 23	19; 10	
	Crawling	9.79 (2.64); 23	19; 9	
	Walking	15.17 (3.48); 28	24; 13	
	Solids	11.24 (4.25); 17	14; 8	
	Words	14.04 (7.08); 24	20; 9	
	Sentences	25.28 (12.08); 21	16; 6	
NBW	Rolling	4.86 (2.20); 269	36; 13	3414.71 (369.67)
	Sitting	6.65 (1.60); 326	47; 14	
	Crawling	8.68 (2.56); 329	46; 15	
	Walking	13.39 (3.63); 455	71; 20	
	Solids	9.07 (3.73); 222	26; 6	
	Words	14.05 (6.89); 346	53; 15	
	Sentences	22.11 (9.80); 299	45; 10	
HBW	Rolling	4.32 (1.73); 48	0; 0	4315.54 (280.37)
	Sitting	6.61 (1.89); 61	0; 0	
	Crawling	8.40 (1.86); 62	0; 0	
	Walking	13.05 (2.75); 85	0; 0	
	Solids	8.87 (3.84); 39	0; 0	
	Words	12.61 (5.23); 64	0; 0	
	Sentences	18.01 (7.74); 49	0; 0	

NOTE: n’s for ELBW and VLBW are 0 because they did not contribute to the analyses.

**Table 7:** Birth weight means and standard deviations for each developmental milestone onset age by gestational age classification considered with birth weight

**Gestational age classification considered with birth weight**	**Developmental milestone**	**Mean onset age in months (standard deviations); n**	**# Premature; # late pre-term (34 to 36 weeks)**	**Means in grams (standard deviations)**
SGA	Rolling	5.08 (2.58); 42	35; 0	2258.47 (582.89)
	Sitting	7.28 (1.90); 51	38; 13	
	Crawling	9.34 (2.35); 47	44; 14	
	Walking	14.30 (4.12); 72	55; 21	
	Solids	10.26 (3.46); 33	25; 9	
	Words	14.73 (7.04); 60	45; 14	
	Sentences	25.68 (11.85); 50	35; 8	
AGA	Rolling	4.79 (2.12); 270	25; 12	3502.65 (357.95)
	Sitting	6.58 (1.53); 325	35; 11	
	Crawling	8.61 (2.53); 295	31; 11	
	Walking	13.34 (3.48); 451	51; 13	
	Solids	9.07 (3.88); 222	21; 5	
	Words	13.99 (6.97); 340	37; 11	
	Sentences	21.79 (9.81); 291	32; 8	
LGA	Rolling	4.58 (1.73); 31	0; 10	4415.12 (304.64)
	Sitting	7.14 (2.18); 40	0; 0	
	Crawling	8.76 (2.02); 39	0; 0	
	Walking	13.17 (2.62); 56	0; 0	
	Solids	8.75 (3.01); 28	0; 0	
	Words	12.50 (4.26); 43	0; 0	
	Sentences	17.84 (7.75); 34	0; 0	

Pearson’s correlations for each birth weight category showed relationships between birth weight and ages at which children achieved independent sitting, walking, and eating solids. Larger birth weights for HBW children was correlated with earlier independent sitting (*r*(61) = −0.42, *p* = .001). Heavier birth weights for NBW was linked with eating solids sooner (*r*(222) = −0.56, *p* = 0.018). Higher birth weights were associated with earlier walking onsets for LBW (*r*(28) = −0.58, *p* = .001) and NBW (*r*(455) = −0.11, *p* = 0.021) children. No significant relationships were found between birth weight category and rolling, crawling, saying words, or speaking sentences (all *p’*s > .05). Birth weight was not correlated with age at evaluation for any developmental milestones (all *p’*s > .05).

We also ran Pearson’s correlations for each gestational age classification considered with birth weight on birth weight and developmental milestone achievement. We found significant relationships between birth weight and rolling, independent sitting, walking, and saying words. Higher birth weights for LGA children was associated with rolling at a younger age (*r*(31) = −0.36, *p* = 0.048) whereas higher birth weights for AGA children was correlated with walking (*r*(451) = −0.12, *p* = 0.01) and saying words (*r*(340) = −0.13, *p* = 0.019) at younger ages. Higher birth weights were also associated with sitting independently at a younger age for SGA (*r*(51) = −0.36, *p* = 0.009) and AGA (*r*(325) = −0.13, *p* = 0.023) children. No relationships were found between gestational age classification considered with birth weight and crawling, eating solids, or saying sentences (all *p’*s > .05). The only significant correlation for age at evaluation was with rolling for LGA infants (*r*(30) = 0.45, *p* = 0.013); older ages at evaluation were associated with rolling later.

## Discussion

The purpose of this study was to examine the possible relationship between birth weight, birth history, and developmental milestone achievement in a retrospective, convenience sample from a clinic population identified as or suspected of having sensory processing disorder. Our results showed that infants with related birth complications who had lower birth weights than infants without birth complications were NBW, AGA, or SGA. Ages of achievement for developmental milestones and birth weight were linked with birth weight and gestational age considered with birth weight category. Earlier ages for independent sitting in HBW infants, eating solids in NBW infants, and walking onsets in LBW and NBW infants were associated with larger birth weights. Rolling at a younger age in LGA infants, earlier walking and speaking words in AGA infants, and sooner independent sitting in SGA and AGA infants were also linked with higher birth weights.

Our results showed that birth history was associated with lower birth weights for AGA, NBW, and SGA infants. We hypothesized that birth history would be linked with lower birth weight for SGA infants because of medical complications (e.g. respiratory distress) requiring professionals to deliver infants expediently. Lower birth weight may have been related to birth history for AGA and NBW infants in our sample because these infants also had complications necessitating a swift delivery that required assistance (e.g. forceps). Also, our sample consisted of children who were being evaluated for sensory processing disorder, so there may have been a higher incidence of birth complications in this sample even for AGA and NBW infants [16].

We found that larger birth weights were associated with earlier onset ages for some developmental milestones. However, the relationship between birth weight and milestone achievement varied according to birth weight category and gestational age classification considered with birth weight. For gestational age classification considered with birth weight, we found that heavier SGA and AGA infants sat earlier and that heavier AGA infants walked and said words earlier. Previous studies have shown that SGA infants tend to have less body fat than LGA infants and some AGA infants [17], and that LGA infants have lower lean body mass than AGA infants [18]. Less body fat in SGA infants and more lean body mass in AGA infants may have allowed them to achieve some motor milestones sooner than the LGA infants in our sample. One of the most difficult tasks for infants is to hold their bodies up against gravity due to uneven body proportions [19,20]. Until 24 months old, infants and toddlers are top heavy, which makes their center of mass high [19,21,22]. During forward progression, a high center of mass requires toddlers to work hard at mitigating the forward movement their center of mass [23,24]. When older children and adults walk, they use active plantarflexion of the stance limb to propel themselves upward. Therefore, they keep the acceleration of their center of mass positive when each foot contacts the ground [23,25,26]. But, toddlers lack adequate muscle strength and coordination to control the forward movement of their center of mass when they walk. Instead, at foot contact, the acceleration of new walkers’ center of mass is negative, which means that they fall downward into their steps [23,25,26]. Therefore, LGA infants may be at a disadvantage for sitting and walking because of high fat and low muscle mass, but at an advantage for rolling because it does not require holding themselves up against gravity. Although we do not have body composition data for these infants, it could be that the HBW and NBW infants in our sample have body composition characteristics (e.g. high muscle mass) that allowed them to achieve earlier sitting and walking. Also, children with sensory processing disorder commonly have low muscle tone, poor postural control and stability, and decreased vestibular functioning [27]. Thus, early motor skills development may be impacted by these factors as well. Increased weight may be an advantage for them, but too much may result in poor milestone development because of the above factors. It is important to further examine the relation of sensory processing disorder to these areas.

Mastering postural control sooner with independent sitting and walking may predispose children to achieving other developmental milestones sooner; we may have found a relationship between birth weight and eating for NBW infants as well as saying words to AGA infants because their ability to be upright (i.e. earlier walking for NBW and earlier sitting and walking for AGA infants) allows for eating solids earlier and for engaging in interactions with others sooner. Meeting motor milestones earlier has been shown to foster meeting other developmental milestones earlier. For instance, the beginning of crawling and walking are associated with increases in verbal [28] and cognitive abilities [29]. The findings about relationships between birth weight and the onset of developmental milestones from this study is important to our understanding of children with sensory processing disorder. In particular, it highlights the need for examining the additive influence of sensory processing disorder in light of children’s development [16].

The limitations to this study are that: 1) the retrospective nature of this study did not allow us to control how many participants were represented in categories based on sex, birth weight category, gestational age classification considered with birth weight, the presence of incomplete histories, or what parents’ exact definition of the onset of milestones were and 2) the fact that we sampled our data from a clinical population may limit the generalizability of our findings, but provides important information on describing this population of children with sensory processing disorder. Future studies involving the collection of prospective data from non-clinical sources are underway to ameliorate these limitations. This study is a first step into examining how variations in birth weight and gestational age considered with birth weight categories relate to birth history and developmental milestone achievement.

## Conclusions

Our findings suggest that birth weight and gestational age considered with birth weight categories provide unique insights into infants’ birth history and developmental milestone achievement. Future studies may help to examine how information about birth weight category and gestational age considered with birth weight can assist health professionals to predict and to treat impairments down the road for children.

## Competing interests

The authors declare that they have no competing interests.

## Authors’ contributions

SG participated in data analyses, project conceptualization, and manuscript writing. TMB and AT participated in project conceptualization and manuscript writing/editing. ES entered, processed, coded, and analyzed participants’ responses. All authors read and approved the final manuscript.

## Pre-publication history

The pre-publication history for this paper can be accessed here:

http://www.biomedcentral.com/1471-2431/13/29/prepub
